# A Case of Viral Myositis

**DOI:** 10.7759/cureus.101032

**Published:** 2026-01-07

**Authors:** Mindy Goh, Charmi Shah

**Affiliations:** 1 Department of Medicine, David Geffen School of Medicine, University of California Los Angeles, Torrance, USA

**Keywords:** general pediatrics, influenza a infection, myalgia, post-viral sequelae, viral myositis

## Abstract

Benign acute childhood myositis (BACM) is a post-viral syndrome characterized by calf pain and tenderness, causing difficulty walking. We present a case of BACM secondary to an acute viral illness, where a 12-year-old boy presented with two weeks of a viral prodrome followed by bilateral calf pain and inability to ambulate. Laboratory evaluation revealed elevated creatine phosphokinase (CPK) and aspartate aminotransferase (AST), as well as leukopenia and thrombocytopenia. Renal function was normal, and a urinalysis was negative for blood or myoglobin. The patient was admitted for intravenous hydration and monitoring. Within 24 hours, his CPK levels returned to normal, the calf pain resolved, and he was safely discharged. Although BACM follows a self-limited course, this case highlights how severe cases may require supportive management in the acute care setting.

## Introduction

Benign acute childhood myositis (BACM) is a transient, self-limited muscular disorder that typically presents in school-aged children after a viral illness [1]. BACM is more commonly seen in late winter and early spring, following the peak of influenza season, reflecting its strong association with viral respiratory infections [1]. The condition is characterized by acute bilateral calf pain, tenderness, and difficulty walking [1]. It is thought to result from a viral infection, triggering both immune-mediated muscle inflammation and a direct muscle injury [1]. The incidence of BACM has not been well established, as most of the literature consists of single-center retrospective studies [1-5]. While evidence suggests that BACM is relatively uncommon, it is an important clinical entity to recognize, as its presentation can mimic more serious neuromuscular, infectious, or metabolic disorders such as Guillain-Barré syndrome or rhabdomyolysis. We present a case of BACM in a 12-year-old boy following co-infection with influenza A and respiratory syncytial virus (RSV), highlighting the typical features, diagnostic approach, clinical management, and rapid resolution characteristic of this condition.

## Case presentation

A 12-year-old boy presented to his primary care physician with a two-week history of cough, nasal congestion, and persistent fevers reaching 103.8°F. He developed acute-onset bilateral calf pain one day prior to presentation, resulting in difficulty ambulating. His medications included daily fluticasone nasal spray and cetirizine for presumed allergic rhinitis, which he has been taking chronically. His parents denied a family history of neuromuscular and autoimmune diseases. He denied recent travel, strenuous exercise, prolonged immobility, rashes, insect bites, gastrointestinal symptoms, urinary symptoms, or decreased oral intake.

On physical examination, the patient exhibited dry mucous membranes and bilateral calf tenderness without swelling, deformity, or bony tenderness. Neurological examination revealed 2+ and symmetric deep tendon reflexes and intact sensation to light touch on the bilateral lower extremities. His muscle bulk and tone were normal. Muscle strength was 5/5 throughout the bilateral upper and lower extremities. The range of motion of the bilateral hips and knees was within normal limits. He was able to bear only minimal weight and exhibited a slow and hesitant gait limited by pain. Laboratory evaluation revealed elevated creatine phosphokinase (CPK) and aspartate aminotransferase (AST) levels (Table 1). Renal function and alanine aminotransferase (ALT) were within normal limits. Given the significantly elevated CPK and difficulty ambulating, he was referred to the emergency department (ED) for further management.

**Table 1:** Laboratory findings throughout the clinical course N/C: not checked.

Test	Reference range	At clinic visit	At hospital admission	At hospital discharge	At post-hospital follow-up
AST (U/L)	13-62	133	164	N/C	N/C
CPK (U/L)	63-473	2,699	3,206	688	175
WBC (x10E^3^/uL)	4.16-9.95	N/C	2.0	N/C	6.13
Platelets (x10E^3^/uL)	143-398	N/C	126	N/C	275

In the ED, repeat laboratory studies demonstrated further elevation of CPK and AST. Complete blood count revealed leukopenia and thrombocytopenia with bandemia (Table 1). Urine myoglobin was negative. A respiratory viral panel was positive for both influenza A and RSV.

**Figure 1:**
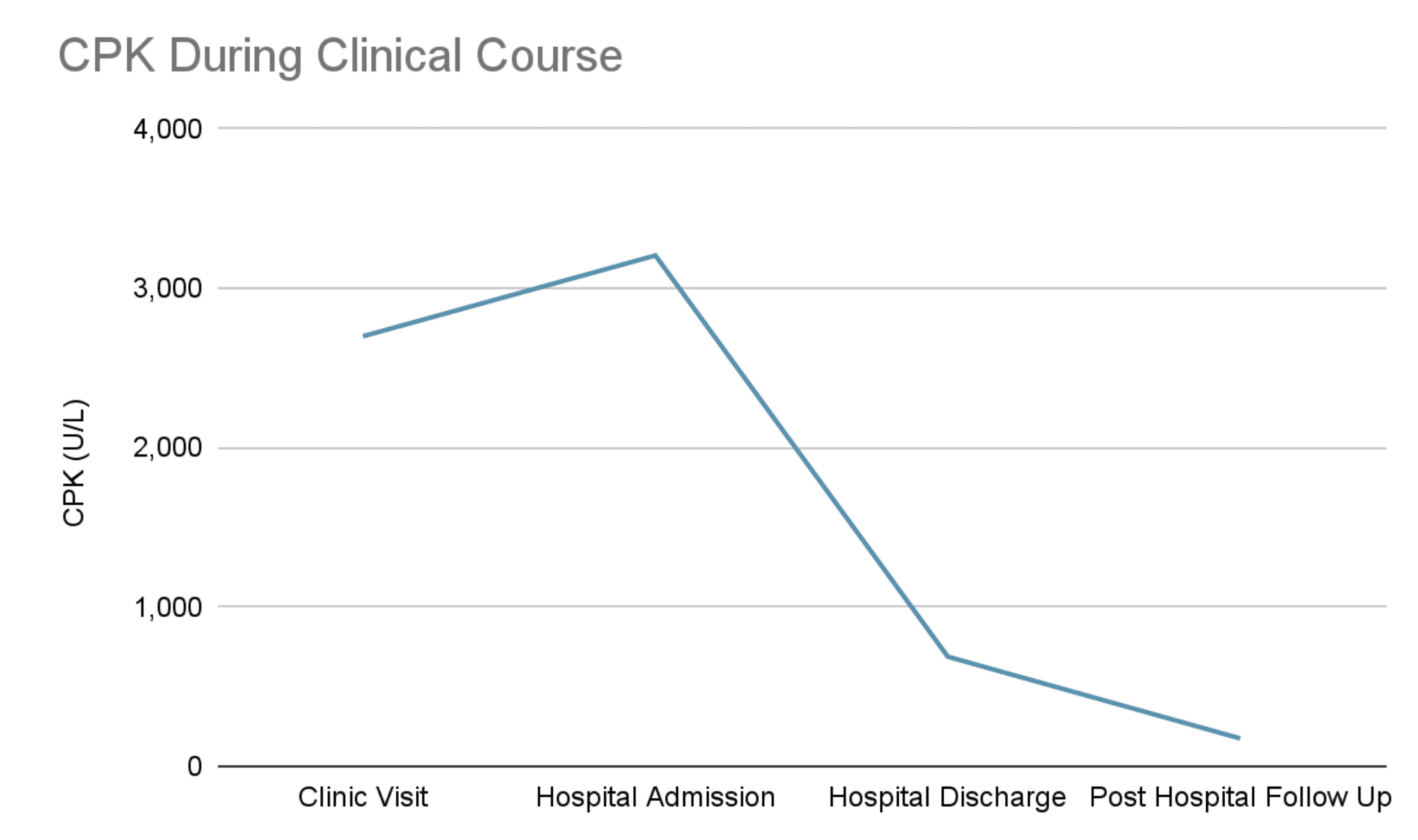
Creatine phosphokinase (CPK) level during the clinical course

The patient was admitted for intravenous fluid therapy, given the progressive CPK elevation and inability to ambulate. He was also given a five-day course of oseltamivir. After 24 hours of aggressive hydration, his CPK decreased to 688 U/L, and he was able to ambulate with minimal pain. He was discharged in stable condition. At his post-hospitalization follow-up four days after discharge, the patient reported further symptomatic improvement, he was back to his functional baseline, and his CPK further downtrended to 175 U/L (Figure 1).

## Discussion

This case illustrates a presentation of BACM, a self-limited post-viral syndrome most commonly seen in school-aged boys, which should be considered in the differential when evaluating a pediatric patient with difficulty walking. Although BACM typically occurs after a brief viral prodrome averaging about five days, this presentation was atypical, with the patient experiencing prodromal symptoms for approximately two weeks prior to the onset of calf pain [2]. The patient presented with acute bilateral calf pain following this prolonged viral prodrome, accompanied by markedly elevated serum CPK, but with otherwise preserved renal function and rapid clinical recovery after supportive management.

BACM typically occurs after viral infections, most commonly influenza A and B, though other viral pathogens such as parainfluenza, adenovirus, and enterovirus have also been implicated. Coinfection with two viruses, such as influenza A and RSV, as seen in our patient, has not been reported in the literature and may account for the longer-than-average prodromal phase observed in this patient [1]. It typically presents with bilateral leg pain, abnormal gait, and fever [1]. Patients with BACM often demonstrate a wide-based, stiff-legged, shuffling gait, commonly referred to as a "Frankenstein gait," but also may toe-walk, or even be non-ambulatory [2]. The affected muscles in the literature have primarily been the gastrocnemius and soleus complex; however, the upper extremities, trunk, and thighs have been implicated as well [3]. Aside from the abnormal gait, patients do not exhibit other abnormal neurological findings, which can help distinguish this illness from a neuromuscular disease process [4]. It is important to note that while myalgia can occur in viral syndromes, it is typically mild and associated with normal CPK levels. In contrast, the muscle pain observed in BACM is often more acute and severe, as demonstrated in this case [5]. The abrupt onset of calf pain, difficulty ambulating, and temporal relationship to a viral illness in this case are consistent with prior case reports [6]. The absence of rash, trauma, recent immobility, and focal neurologic deficits further helped narrow the differential diagnosis. The lack of neurologic weakness or sensory changes argued against Guillain-Barré syndrome and other polyneuropathies. The patient did not have a history of trauma, localized swelling, or unilateral calf involvement, making deep vein thrombosis or traumatic muscle injury unlikely. The absence of cutaneous findings reduced concern for inflammatory myopathies associated with dermatologic manifestations, such as dermatomyositis. Collectively, these findings supported a diagnosis of BACM, particularly in the setting of recent viral illness and isolated calf pain with elevated CPK.

BACM is diagnosed based on the overall clinical picture and supportive laboratory findings. Laboratory evaluation in this patient demonstrated a markedly elevated CPK, often seen in cases of BACM. CPK levels can range from several hundred to more than 10,000 U/L in reported cases, with levels typically peaking within the first few days of presentation and normalizing within a week [4]. The mild elevation in AST is also frequently reported, as it may originate from muscle injury rather than hepatic dysfunction [5]. In other cases, elevated levels of lactic dehydrogenase have also been reported [5]. Laboratory findings associated with preceding viral infection can also be seen in cases of BACM, such as the leukopenia and thrombocytopenia present in our patient.

Management of BACM is largely supportive, focusing on hydration to minimize the risk of renal complications and pain management. A scoping review of BACM cases revealed that renal complications were rare, suggesting that supportive treatment at home is likely adequate if the patient can reliably hydrate [6]. However, hospitalization may be warranted in patients with severe pain, inability to ambulate, and inability to keep up with aggressive oral hydration, as seen in this patient. With supportive care, patients usually recover within a week [6]. Antiviral medications can be help shorten the duration of other viral symptoms if initiated early in the disease course, but their utility in treating the myositis is unclear. A general review of myositis by Crum-Cianflone in 2008 notes that the “efficacy of antiviral treatment for acute benign myositis is unknown and hence is not currently recommended,” primarily due to the onset of myositis later in the disease course, when antivirals already yield little benefit [6]. On the other hand, a retrospective study by Turan et al. showed that patients with BACM who received oseltamivir recovered one day sooner than those who did not, a difference found to be statistically significant [7].

In this case, the patient’s inability to bear weight and his uptrending CPK prompted inpatient admission and intravenous fluid administration. The subsequent rapid downtrend in CPK and complete recovery of ambulation within 24 hours are consistent with the benign course described in other cases.

## Conclusions

BACM is a self-limited illness that can occur in pediatric patients after a viral illness. The clinical presentation commonly consists of sudden-onset bilateral calf pain and difficulty ambulating after a viral prodrome, as seen in our patient. While most patients fully recover with supportive care at home, patients with more severe cases may have markedly elevated CPK levels and may require hospitalization for more aggressive intravenous hydration like our patient. Notably, patients have an unremarkable neurologic exam, which helps distinguish this illness from neuromuscular conditions.
